# FOX-2 Dependent Splicing of Ataxin-2 Transcript Is Affected by Ataxin-1 Overexpression

**DOI:** 10.1371/journal.pone.0037985

**Published:** 2012-05-30

**Authors:** Franziska Welzel, Christian Kaehler, Melanie Isau, Linda Hallen, Hans Lehrach, Sylvia Krobitsch

**Affiliations:** 1 Otto Warburg Laboratory, Max Planck Institute for Molecular Genetics, Berlin, Germany; 2 Department of Vertebrate Genomics, Max Planck Institute for Molecular Genetics, Berlin, Germany; 3 Department of Biology, Chemistry and Pharmacy, Free University Berlin, Berlin, Germany; University of Florida, United States of America

## Abstract

Alternative splicing is a fundamental posttranscriptional mechanism for controlling gene expression, and splicing defects have been linked to various human disorders. The splicing factor FOX-2 is part of a main protein interaction hub in a network related to human inherited ataxias, however, its impact remains to be elucidated. Here, we focused on the reported interaction between FOX-2 and ataxin-1, the disease-causing protein in spinocerebellar ataxia type 1. In this line, we further evaluated this interaction by yeast-2-hybrid analyses and co-immunoprecipitation experiments in mammalian cells. Interestingly, we discovered that FOX-2 localization and splicing activity is affected in the presence of nuclear ataxin-1 inclusions. Moreover, we observed that FOX-2 directly interacts with ataxin-2, a protein modulating spinocerebellar ataxia type 1 pathogenesis. Finally, we provide evidence that splicing of pre-mRNA of ataxin-2 depends on FOX-2 activity, since reduction of FOX-2 levels led to increased skipping of exon 18 in ataxin-2 transcripts. Most striking, we observed that ataxin-1 overexpression has an effect on this splicing event as well. Thus, our results demonstrate that FOX-2 is involved in splicing of ataxin-2 transcripts and that this splicing event is altered by overexpression of ataxin-1.

## Introduction

Alternative splicing is a key posttranscriptional mechanism controlling the expression of numerous gene products contributing to proteome diversity in a cell- and tissue-specific manner [1], [2], [3]. Variations in the intracellular concentration and localization of splicing factors influence alternative splicing and may be a natural mechanism for regulating gene expression [4]. Aberrant splicing perturbs this normal balanced functionality and is related to a number of human disorders causing disease directly or mediating disease modification and susceptibility, as disease genes have the propensity to be more intron-rich, and therefore are more susceptible to splicing deregulation [1], [3], [5].

Of note, a large group of RNA binding proteins regulating pre-mRNA splicing have been identified, but for most the precise splicing events are barely understood. To a certain extent the splicing activities of the RNA binding proteins FOX-1 and FOX-2, members of the FOX family (feminizing gene on X), have been revealed (reviewed in [6]). Both splicing regulators are conserved across mammalian genomes and highly expressed in brain, heart and muscle. Moreover, FOX-1 and FOX-2 are highly homologous, as both contain a conserved RNA recognition motif (RRM) in the central region, which is responsible for binding the UGCAUG motif of target RNAs, whereas the N- and C-terminal regions are less conserved. Due to the use of tissue-specific promoters and alternative splicing, several FOX variants have been identified, some of these have a specific C-terminal sequence RF(A/T)PY, which likely serves as a conserved nuclear localization signal of this protein family (reviewed in [6]). In contrast, FOX variants with different C-terminal endings are found to be predominantly cytoplasmic potentially leading to different cellular functions [7], [8].

Interestingly, a global computational prediction utilizing the FOX-binding motif UGCAUG resulted in a defined regulatory splicing network, which comprises more than thousand FOX-1/FOX-2 targets with enrichment in genes implicated in neuromuscular processes [9]. Of note, most of these target genes have been linked to various human genetic disorders, such as neurological, neurodegenerative or heart disorders amongst others, or are encoding splicing factors *per se* [6], [9], [10]. Depending upon the presence of this motif in upstream or downstream intronic flanking regions, binding of FOX-1 or FOX-2 represses or enhances exon inclusion, respectively (reviewed in [6]). Besides, mutations in FOX-1 or its abnormal expression have been found in patients with epilepsy, mental retardation, autism and heart disease [11], [12], [13]. FOX-2 has been found differentially spliced in breast cancer cells and was identified as a novel hub gene in a colon cancer-specific gene network [14], [15]. Interestingly, both FOX-1 and FOX-2, also known as A2BP1 (ataxin-2 binding protein 1) and RBM9 (RNA binding motif protein 9), have been identified as interaction partners of ataxin-1 (ATXN1), which is implicated in spinocerebellar ataxia type 1 (SCA1) [16]. This disease belongs to the family of so-called polyglutamine disorders that further comprises Huntington's disease, spinobulbar muscular atrophy, dentatorubral pallidoluysian atrophy, and spinocerebellar ataxia type 2, 3, 6, 7 & 17. All usually strike in late midlife and are characterized by progressive neuronal dysfunction and loss of specific neuronal populations. Causative for these disorders is an expansion of the trinucleotide repeat CAG in otherwise unrelated genes encoding an enlarged polyglutamine region in the disease-causing proteins, for which the cellular functions are yet not clearly understood [17], [18], [19]. The gene causative for SCA1 has been cloned and identified on chromosome 6p23 and termed *ATXN1* [20], [21]. Normal *ATXN1* alleles comprise 4–39 CAG repeats, whereas alleles of SCA1 patients comprise 40–83 CAG repeats leading to an expanded polyglutamine region in the N-terminal part of ATXN1 [22], [23], [24]. This mutant protein accumulates in nuclear inclusions that are detected in post-mortem brain tissue of SCA1 patients and transgenic SCA1 mouse models representing a neuropathological hallmark of SCA1 [22], [24]. Moreover, these nuclear ATXN1 inclusions are the source for the recruitment of other cellular proteins and aberrant protein-protein interactions thereby contributing to cellular dysfunction [25], [26], [27], [28], [29], [30].

Interestingly, an interaction between FOX-1 and ataxin-2 (ATXN2), the disease protein in another SCA, spinocerebellar ataxia type 2 (SCA2), has been reported as well [16], [31]. Notably, interactions between FOX-1 and FOX-2 and other ataxia-causing proteins have been identified, indicating that both splicing factors are part of a main hub in a human ataxia protein network [16]. Thus, some of the human inherited ataxias might represent RNA splicing disorders as proposed or demonstrated for other neurodegenerative and neurological disorders, since alternative splicing is particularly prevalent in the brain [16], [32]. This is further supported by the finding that genes implicated in RNA binding and processing modify neurodegeneration in transgenic fly models of polyglutamine disorders [33], [34].

In this study we set out to functionally characterize the interaction between FOX-2 and ATXN1 that has been originally identified in a high-throughput yeast-2-hybrid analysis [16], and the effect of ATXN1 overexpression on FOX-2 localization and splicing activity.

## Results

### Interaction between ATXN1 and FOX-2 variants

**Figure 1:**
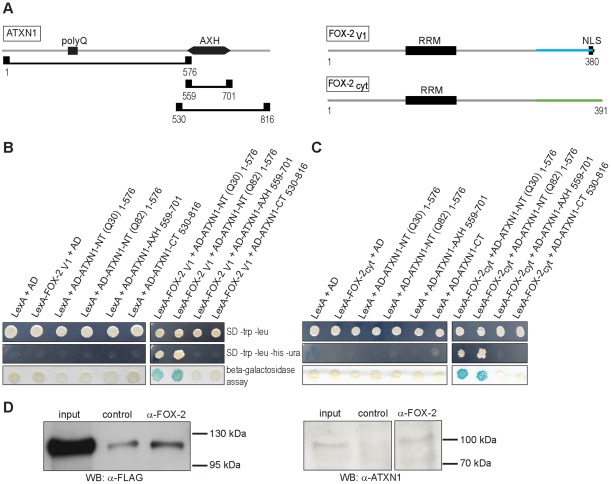
ATXN1 interacts with FOX-2 splice variants. (**A**) Schematic view of ATXN1 and the regions used in the Y2H analyses (left) and FOX-2 variants (right). Prey plasmids pACT-ATXN1-NTQ30 or pACT-ATXN1-NTQ82 cover amino acids 1–576, pACT-ATXN1-AXH amino acids 559–701, and pACT-ATXN1-CT amino acids 530–816. Bait plasmid pBTM-FOX-2_V1_ covers amino acids 1–380 of variant 1, including the putative NLS in the C-terminal region, and pBTM-FOX-2_cyt_ covers amino acids 1–391 of the cytoplasmic FOX-2 variant. Different C-terminal regions of both FOX-2 variants are highlighted in blue and green. (**B; C**) For directed Y2H analyses yeast strain L40ccua was co-transformed with the relevant bait and prey plasmids, and transformants were selected and spotted onto selective media or membrane to analyze activity of the reporter genes. (**D**) For Co-IP experiments, HEK293T cell lysates derived from cells overexpressing FLAG-ATXN1-Q30 (left panel) or HEK293T cell lysates (right panel) were incubated with an antibody directed against FOX-2 (Bethyl or Abnova, respectively). Cell lysates incubated without primary antibody served as controls. Then, membranes were treated with an antibody directed against the FLAG tag or ATXN1 to detect precipitated protein.

As mentioned before, FOX-2 has been shown to be part of a main hub found in a protein-protein interaction network for ataxia-causing proteins that have been implicated in more than 20 different inherited cerebellar ataxias [16]. Interestingly, ATXN1 has been identified as one interaction partner of FOX-2 [16]. To further characterize this interaction, we first carried out directed yeast-2-hybrid (Y2H) analyses. For this, we generated different prey constructs for ATXN1, which are schematically shown in Fig. 1A (left panel). The plasmids pACT-ATXN1-NTQ30 and pACT-ATXN1-NTQ82 encode the N-terminal region of ATXN1, which comprises the polyglutamine region with 30 or 82 glutamines, representing normal and disease state, respectively. Plasmid pACT-ATXN1-AXH encodes the AXH-domain of ATXN1, an RNA-binding motif, which is implicated in the self-association of ATXN1 [35]. Furthermore, the transcriptional repression activity of ATXN1 relies on this domain [36]. Plasmid pACT-ATXN1-CT encodes the C-terminal portion of the protein, including the AXH-domain. On the other hand, we generated bait constructs for expression of a predominantly nuclear and cytoplasmic FOX-2 variant, termed FOX-2_V1_ and FOX-2_cyt_, respectively, which are schematically shown in Fig. 1A (right panel; please see Material and Methods and Fig. S1 for further details). The rationale behind this is based on the fact that use of alternative promoters and alternative splicing patterns generate diverse FOX protein variants with different N- or C-terminal regions that do not preferentially localize to the nucleus but also to the cytoplasm [7], [8], [37]. Both variants used show differences exclusively in the C-terminal region resulting in the loss of the putative nuclear localization signal in the variant FOX-2_cyt_ (Fig. S1A). Moreover, we decided to make use of both FOX-2 variants, since ATXN1 has been found to shuttle between the nucleus and the cytoplasm [25]. First of all we performed Y2H analyses to exclude the possibility that the bait proteins LexA-FOX-2_V1_ and LexA-FOX-2_cyt_ as well as the ATXN1 prey proteins *per se* led to the activation of the analyzed reporter genes. First, bait plasmids pBTM-FOX-2_V1_ and pBTM-FOX-2_cyt_ encoding fusion proteins of the DNA binding domain LexA and the FOX-2 variants, and the prey vector pACT encoding the activation domain (AD) were co-transformed. Secondly, the bait vector pBTM encoding the DNA binding domain LexA was co-transformed with the prey constucts pACT-ATXN1-NTQ30, pACT-ATXN1-NTQ82, pACT-ATXN1-AXH, or pACT-ATXN1-CT encoding the fusion proteins AD-ATXN1-NT(Q30)_1–576_, AD-ATXN1-NT(Q82)_1–576_, AD-ATXN1-AXH_559–701_, or AD-ATXN1-CT_530–816_, respectively_._ Consequently, the respective yeast transformants were isolated and analyzed for reporter gene activity. Reporter gene activity was not observed for all the combinations tested (Fig. 1B and Fig. 1C). In the second step, we performed directed Y2H analyses to test for a potential interaction between the two FOX-2 variants and ATXN1. We observed that yeast cells expressing LexA-FOX-2_V1_ and AD-ATXN1-NT(Q30)_1–576_ or AD-ATXN1-NT(Q82)_1–576_ exhibited activity of the reporter genes (Fig. 1B). Interestingly, activity of reporter genes was also detected for yeast cells expressing LexA-FOX-2_cyt_ and AD-ATXN1-NT(Q30)_1–576_ or AD-ATXN1-NT(Q82)_1–576_ (Fig. 1C). Reporter gene activity was not observed for yeast cells expressing LexA-FOX-2_V1_ or LexA-FOX-2_cyt_ and AD-ATXN1-AXH_559–701_ or AD-ATXN1-CT_530–816_, respectively. We therefore concluded that an interaction between both FOX-2 variants and the N-terminal region of ATXN1 occurs in the Y2H system.

To further validate the interaction between FOX-2 and ATXN1, we performed co-immunoprecipitation (Co-IP) experiments using lysates prepared from HEK293T cells overexpressing FLAG-ATXN1-Q30 or lysates from non-transfected HEK293T cells for the detection of endogenous interactions. As shown in Fig. 1D (left panel), FLAG-ATXN1-Q30 protein was precipitated with an antibody directed against FOX-2. A minimal amount of protein was precipitated in the control sample lacking primary antibody. In addition, endogenous ATXN1 was enriched with an antibody directed against FOX-2 that recognizes a number of FOX-2 protein variants as well (Fig. 1D, right panel). Thus, ATXN1 is found in association with FOX-2 in mammalian cells.

### Nuclear ATXN1 inclusions affect FOX-2 localization

**Figure 2:**
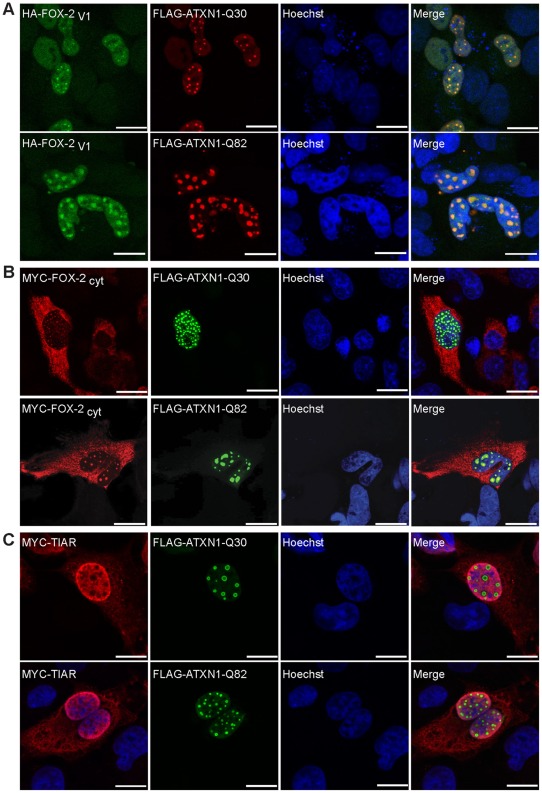
The FOX-2 splice variants FOX-2_V1_ and FOX-2_cyt_ co-localize with nuclear ATXN1 inclusions. Confocal microscopy of HeLa cells transfected with (**A**) pCMV-HA-FOX-2_V1_ and pcDNA1-FLAG-SCA1-Q30 or pcDNA1-FLAG-SCA1-Q82, or (**B**) pCMV-MYC-FOX-2_cyt_ and pcDNA1-FLAG-SCA1-Q30 or pcDNA1-FLAG-SCA1-Q82, and (**C**) pCMV-MYC-TIAR and pcDNA1-FLAG-SCA1-Q30 or pcDNA1-FLAG-SCA1-Q82, respectively. Forty-eight hours post transfection cells were fixed and prepared for microscopic analyses. Proteins were visualized using the respective antibodies against the tag as described in Material and Methods. Nuclei were stained using Hoechst. Bars represent 20 µm.

To further confirm that the protein-protein interaction between FOX-2 and ATXN1 is valid, we next carried out localization studies. The important aspect here is that ATXN1 has been reported to be predominantly nuclear, however shuttling of ATXN1 between nucleus and cytoplasm has been reported [25], [38]. In line with this, we observed in our Y2H analyses that ATXN1 is able to interact with the mainly cytoplasmic FOX-2_cyt_ splice variant as well. HeLa cells were co-transfected with the mammalian expression plasmids pcDNA1-FLAG-SCA1-Q30 or pcDNA1-FLAG-SCA1-Q82 to express full-length ATXN1 with 30 or 82 consecutive glutamines and pCMV-HA-FOX-2_V1_ or pCMV-MYC-FOX-2_cyt_, respectively. Afterwards, cells were prepared for confocal microscopy as described in Material and Methods. We observed that the protein HA-FOX-2_V1_ displayed a predominantly diffuse nuclear localization, whereas MYC-FOX-2_cyt_ was evenly distributed in the cytoplasm (Fig. S1B). Moreover, we detected that in cells overexpressing the proteins FLAG-ATXN1-Q30 or FLAG-ATXN1-Q82 the variant HA-FOX-2_V1_ co-localized with the nuclear inclusions formed by these proteins (Fig. 2A). These nuclear inclusions, which are a pathological hallmark in SCA1, are heterogeneous and display diverse body morphology [22], [24]. We observed that co-localization of FOX-2 was more prominent with larger inclusions, but co-localization was also observed with smaller inclusions formed by FLAG-ATXN1-Q30 and FLAG-ATXN1-Q82. Interestingly, the cytoplasmic MYC-FOX-2_cyt_ variant co-localized with both larger and smaller nuclear ATXN1 inclusions as well (Fig. 2B). To further investigate the specificity of this co-localization, we included the protein TIAR (TIA1 cytotoxic granule-associated RNA binding protein-like 1), a splicing regulator comprising RRM domains like FOX-2 [39], [40]. For this, HeLa cells were co-transfected with the mammalian expression plasmids pcDNA1-FLAG-SCA1-Q30 or pcDNA1-FLAG-SCA1-Q82 and pCMV-MYC-TIAR and prepared as described. In this case, we observed that the presence of nuclear ATXN1 inclusions had no effect on the localization of TIAR (Fig. 2C), indicating that the mis-localization of FOX-2 noticed in the presence of nuclear ATXN1 inclusions is specific, and is not based on unspecific trapping effects of RNA binding proteins.

**Figure 3:**
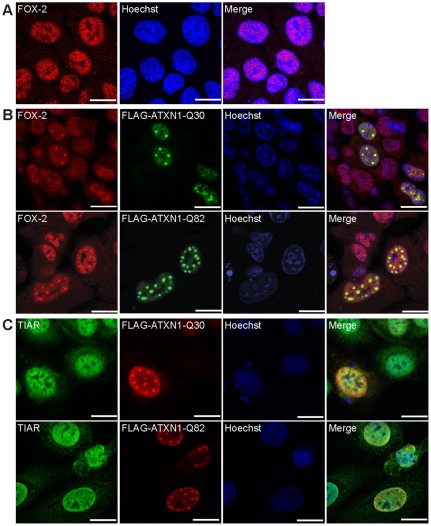
FOX-2 co-localizes with nuclear ATXN1 inclusions. (**A**) Endogenous localization of FOX-2 in HeLa cells visualized with an antibody directed against FOX-2 (Bethyl). (**B; C**) HeLa cells expressing normal and mutant ATXN1 were fixed forty-eight hours post transfection. Endogenous proteins were visualized using respective antibodies against (**B**) FOX-2 (Bethyl) and FLAG or (**C**) TIAR and FLAG as described. Nuclei were stained using Hoechst. Bars represent 20 µm.

Furthermore, we investigated whether the observed mis-localization of FOX-2 in the presence of ATXN1 inclusions holds true for endogenous FOX-2 protein. Again, HeLa cells were transfected with expression plasmids pcDNA1-FLAG-SCA1-Q30 or pcDNA1-FLAG-SCA1-Q82. Of note, we observed that the localization of endogenous FOX-2 protein (Fig. 3A) was affected, since it co-localized with nuclear ATXN1 inclusions as well (Fig. 3B). Once more, co-localization of FOX-2 seemed to be more prominent with larger nuclear ATXN1 inclusions. To further validate this observation in another cell line, we used HEK293T cells. In addition to HeLa cells, this cell line has been used to study formation of ATXN1 inclusions as well as ATXN1-induced cell death. Moreover, it was demonstrated that both cell lines reproduce molecular mechanisms contributing to SCA1 pathogenesis in the cerebellum [25], [27], [41], [42]. Accordingly, we transfected HEK293T cells with expression plasmids pcDNA1-FLAG-SCA1-Q30 or pcDNA1-FLAG-SCA1-Q82 and prepared the cells for microscopy. As observed in HeLa cells, endogenous FOX-2 co-localized with nuclear inclusions formed by FLAG-ATXN1-Q30 and FLAG-ATXN1-Q82 (Fig. S2). Thus, overexpression of normal and mutant ATXN1 caused mis-localization of endogenous FOX-2 proteins under the chosen experimental settings. Again, to further validate the specificity of this mis-localization, we also analyzed the localization of endogenous TIAR protein in the presence of nuclear ATXN1 inclusions. As shown in Fig. 3C, we observed that nuclear ATXN1 inclusions had no effect on nuclear localization of TIAR under the chosen conditions.

**Figure 4:**
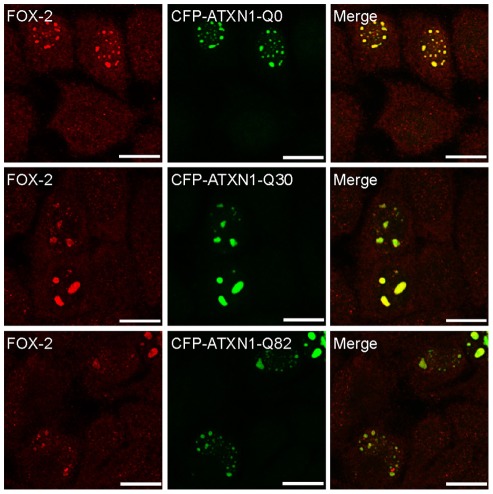
Co-localization of FOX-2 with nuclear ATXN1 inclusions is independent of the polyglutamine region. Confocal microscopy of HeLa cells transfected with CFP-ATXN1-Q0, CFP-ATXN1-Q30 or CFP-ATXN1-Q82, respectively. Forty-eight hours post transfection cells were fixed and prepared for microscopic analyses. FOX-2 protein was visualized using a specific antibody (Abnova). CFP fluorescence was pseudo-coloured green. Nuclei were stained using Hoechst. Bars represent 20 µm.

In the next step, we wanted to investigate if the polyglutamine region is important for the mis-localization of FOX-2. Here we took advantage of the plasmids Tsai and colleagues generated, encoding ATXN1 lacking the polyglutamine stretch or comprising 30 or 82 consecutive glutamines fused to the cyan fluorescent protein [43]. HeLa cells were transfected with the respective constructs and CFP-ATXN1 and FOX-2 localization was examined by confocal microscopy. As described by Tsai and colleagues, overexpression of CFP-ATXN1-Q0, CFP-ATXN1-Q30 and CFP-ATXN1-Q82 led to the formation of nuclear inclusions (Fig. 4). In all cases prominent co-localization of FOX-2 with larger ATXN1 inclusions was detected, demonstrating that the polyglutamine region within ATXN1 is not required for the observed co-localization between FOX-2 and ATXN1.

### FOX-2 splicing activity is affected in the presence of nuclear ATXN1 inclusions

**Figure 5:**
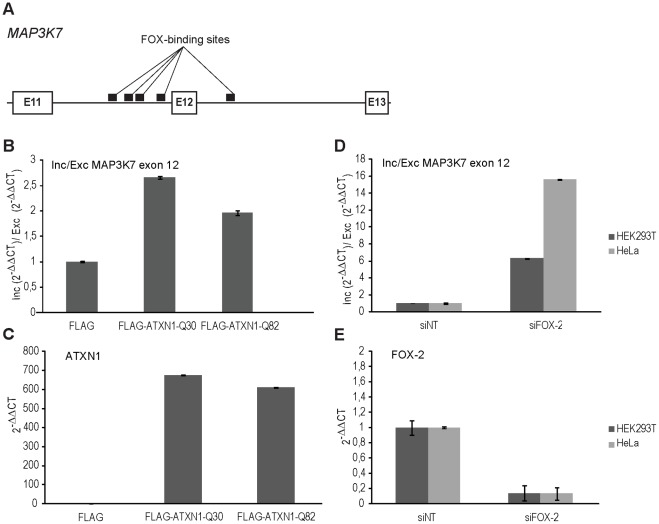
ATXN1 overexpression affects FOX-2 splicing activity. (**A**) Schematic view of a partial genomic region of the *MAP3K7* gene. Exons are represented by white boxes, the intronic regions by a black line. The FOX-binding sites are shown as black rectangles. (**B; C**) Total RNA was isolated from HEK293T cells transiently overexpressing ATXN1 with 30 or 82 glutamines. (**B**) Splicing of the MAP3K7 exon 12 was analyzed by RT-qPCR. Inclusion/exclusion ratio of MAP3K7 exon 12 is illustrated. (**C**) ATXN1 overexpression was analyzed by RT-qPCR. ATXN1 transcript levels detected in the vector control were set as value one. (**D; E**) Total RNA was isolated from HEK293T and HeLa cells that were either transfected with FOX-2-specific siRNA or with control siRNA and splicing of the MAP3K7 exon 12 was analyzed by RT-qPCR. (**D**) Inclusion/exclusion ratio of MAP3K7 exon 12 is demonstrated. (**E**) FOX-2 reduction was analyzed by RT-qPCR in HeLa and HEK293T cells. Error bars indicate standard error of the mean.

Since mis-localization of FOX-2 was observed in the presence of nuclear ATXN1 inclusions, we investigated next whether ATXN1 overexpression has an effect on FOX-2 splicing activity. Here, we focused on one target gene of FOX-2 dependent splicing, *MAP3K7* (mitogen-activated protein kinase kinase kinase 7) (Fig. 5A), as it has been reported that FOX-2 depletion results in increased inclusion of exon 12 in the MAP3K7 transcript [10]. After transfection of HEK293T cells with plasmids pcDNA1-FLAG-ATXN1-Q30 or pcDNA1-FLAG-ATXN1-Q82 or with siRNA molecules for knock-down of FOX-2 and respective controls, cells were lysed, RNA was extracted and RT-qPCR performed as described. Notably, we observed an increase of MAP3K7 transcript with inclusion of exon 12 when either normal or mutant ATXN1 were overexpressed compared to the vector control, increasing the ratio between inclusion and exclusion of MAP3K7 exon 12 by 2.7- or 2.0-fold, respectively (Fig. 5B). Here, we would like to point out that variations in ATXN1 transcript levels of normal and mutant ATXN1 could be accountable for the observed difference in the ratio between inclusion and exclusion of MAP3K7 exon 12 (Fig. 5C). As control, we also analyzed the effect of ATXN1 overexpression on FOX-2 transcription. We did not detect any alterations under the chosen conditions (data not shown). Moreover, a 6.3- or 15.6-fold increase in the ratio between inclusion and exclusion of MAP3K7 exon 12 was also observed for FOX-2 knock-down in HEK293T and HeLa cells, respectively (Fig. 5D). The transcript level of FOX-2 was reduced to approximately 20% (Fig. 5E). Thus, FOX-2 dependent splicing is affected by ATXN1 overexpression.

### Interaction of FOX-2 splice variants with ATXN2

**Figure 6:**
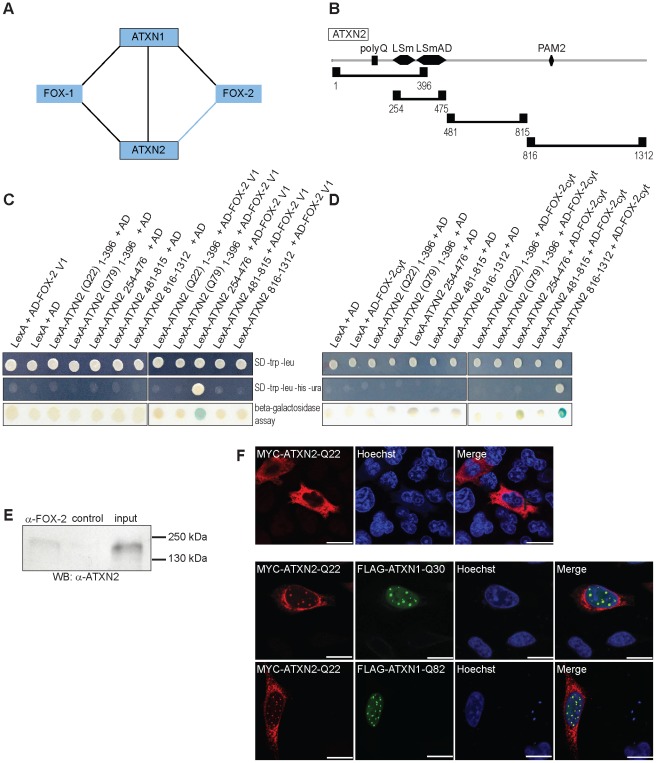
ATXN2 interacts with FOX-2 splice variants and co-localizes with nuclear ATXN1 inclusions upon overexpression. (**A**) Schematic illustration of ATXN1 interactions. Black lines represent interactions reported by Lim and co-workers [16], the blue line represents the investigated interaction in this study. (**B**) Schematic view of ATXN2 regions used in Y2H studies as described earlier [44], [51], [66]. (**C; D**) L40ccua yeast cells expressing the corresponding LexA-ATXN2 and AD-FOX-2 fusion proteins were spotted onto selective media or membrane as indicated and the activity of the reporter genes was monitored. (**E**) HeLa cell lysates were incubated with an antibody directed against FOX-2 (Bethyl) and membranes were treated with an anti-ATXN2 antibody (BD-Biosciences) to detect precipitated protein. (**F**) HeLa cells were transfected with pCMV-MYC-ATXN2-Q22 or co-transfected with pCMV-MYC-ATXN2-Q22 and pcDNA1-FLAG-SCA1-Q30 or pcDNA1-FLAG-SCA1-Q82, respectively. Forty-eight hours post transfection cells were fixed and prepared for microscopic analyses. Nuclei were stained using Hoechst. Bars represent 20 µm.

In addition to the interaction between ATXN1 and FOX-2, an interaction between the FOX-2 paralog, FOX-1 (A2BP1), ATXN1 and ATXN2 has been reported [16] (Fig. 6A). Based on this, we decided to investigate whether ATXN2 and FOX-2 are also found in association. First, we carried out directed Y2H analyses using both FOX-2 variants and ATXN2 regions as indicated in Fig. 6B. Again, we first excluded that the bait LexA-ATXN2 fusion proteins and prey AD-FOX-2 fusion proteins *per se* lead to the activation of the reporter genes by co-transformation of the respective bait and prey plasmids. Reporter gene activity was not detected (Fig. 6C; D). Yeast cells expressing the fusion proteins LexA-ATXN2_254–476_ and AD-FOX-2_V1_ (Fig. 6C) exhibited activity of reporter genes as well as yeast cells expressing LexA-ATXN2_816–1312_ and AD-FOX-2_cyt_ (Fig. 6D)_,_ indicating a direct interaction between FOX-2 variants and ATXN2 as well. Interestingly, we did not detect an interaction between the fusion proteins LexA-ATXN2(Q22)_1–396_ or LexA-ATXN2(Q79)_1–396_, which comprise the N-terminal region of ATXN2 containing the polyglutamine stretch, and FOX-2 variants suggesting that the polyQ region is not critical for this interaction. In addition, Co-IP experiments were carried out, demonstrating that endogenous ATXN2 was precipitated from HeLa cell lysate using an antibody against FOX-2 (Fig. 6E). Thus, besides the interaction with ATXN1, FOX-2 is also found in association with ATXN2.

Since an accumulation of ATXN2 in cells with nuclear ATXN1 inclusions has been observed in a transgenic SCA1 fly model as well as in human SCA1 neurons [28], we finally carried out co-localization studies to investigate whether ATXN2 co-localizes with ATXN1 in the cell line used. We observed that HeLa cells overexpressing ATXN2 in combination with normal or mutant ATXN1 proteins exhibited co-localization of ATXN2 with ATXN1 inclusions as well (Fig. 6F).

In addition, ATXN2 but also a number of RNA-binding proteins with RRM-domains are part of cytoplasmic stress granules under conditions of cellular stress [44], [45]. Therefore, we wanted to further investigate the localization of both FOX-2 splice variants under such conditions. For this, HeLa cells were transiently transfected with expression vectors encoding HA-FOX-2_V1_ or MYC-FOX-2_cyt_, treated with arsenite and prepared for microscopic analysis as described. As shown in Fig. S3A, both FOX-2 variants accumulated in cytoplasmic foci in arsenite-treated cells that co-localized with ATXN2, indicating that both FOX-2 splice variants are part of stress granules. Furthermore, we also monitored endogenous FOX-2 protein localization in HeLa cells. We observed that FOX-2 exhibited a predominantly nuclear localization in untreated and arsenite-treated cells (Fig. S3B). However, quite a few FOX-2 positive cytoplasmic foci were observed in arsenite-treated cells that are also positive for ATXN2 and for another stress granule marker protein analyzed, TIAR [45], [46], further demonstrating that FOX-2 is indeed a component of stress granules.

### FOX-2 regulates splicing of ATXN2 transcripts

**Figure 7:**
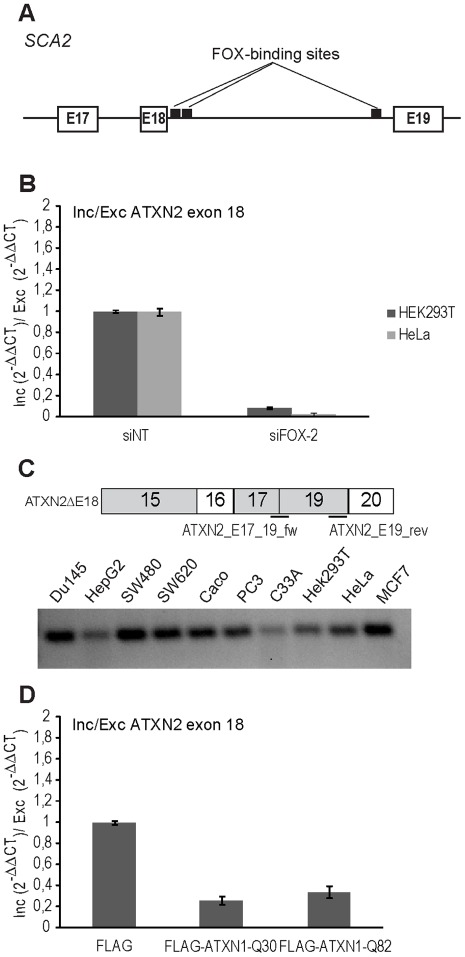
Splicing of ATXN2 transcripts is affected by both, reduced FOX-2 levels and ATXN1 overexpression. (**A**) Schematic view of a partial genomic region of the *SCA2* gene. Exons are represented by white boxes, the respective intronic regions by a black line. The two potential FOX-binding sites 40 bp downstream of exon 18 are shown as black rectangles. (**B**) HEK293T and HeLa cells were either transfected with FOX-2-specific siRNA or with control siRNA. Subsequently, total RNA was isolated and RT-qPCR experiments were performed as described in Material and Methods. Inclusion/exclusion ratio of ATXN2 exon 18 is shown. Error bars indicate standard error of the mean. (**C**) Schematic view of a region of ATXN2 transcripts lacking exon 18 (upper panel) and the primers used for its detection in various cell lines as indicated (lower panel). (**D**) HEK293T cells were transfected with pcDNA1-FLAG-SCA1-Q30 or pcDNA1-FLAG-SCA1-Q82 and processed as described in (**B**).

Remarkably, ATXN2 has been described as a modulator of SCA1 pathogenesis [28]. Therefore it is quite interesting that the *SCA2* gene bears two putative FOX-binding sites ∼ 30–100 nucleotides downstream of exon 18 in the ATXN2 transcript (Fig. 7A) [9], suggesting that FOX-2 could potentially be involved in ATXN2 pre-mRNA splicing. To investigate this, we decided to perform RNAi experiments and to analyze the effect of FOX-2 depletion on splicing of exon 18 of the ATXN2 transcript. After transfecting HEK293T or HeLa cells with siRNA molecules specific for FOX-2 or with control siRNA, RNA was isolated and RT-qPCR performed. As shown in Fig. 7B, reduced levels of FOX-2 led to an enrichment of ATXN2 transcript lacking exon 18 thereby decreasing the ratio between inclusion/exclusion of exon 18 in ATXN2 transcripts in both cell lines to 0.086-fold or 0.026-fold in comparison to controls, demonstrating that FOX-2 is implicated in ATXN2 pre-mRNA splicing. To further confirm the occurrence of this splice variant, which seems to have a relatively low abundance as suggested by the RT-qPCR experiments, we analyzed a panel of cell lines for the existence of ATXN2 transcripts lacking exon 18 with the described primer pair and obtained respective amplicons, of which one was validated by sequencing (Fig. 7C). Interestingly, skipping of exon 18 of ATXN2 transcripts leads to a premature stop codon. This would result in a different C-terminal region, and such a variant has been annotated as protein-coding in ENSEMBL (ENST00000550104).

Consequently, we also investigated the influence of ATXN1 overexpression, since we had observed an effect on splicing activities of FOX-2. For this, HEK293T cells were transiently transfected with plasmids pcDNA1-FLAG-SCA1-Q30 or pcDNA1-FLAG-SCA1-Q82, or an empty vector as control, RNA was isolated and RT-qPCR experiments were performed. We observed that overexpression of FLAG-ATXN1-Q30 or FLAG-ATXN1-Q82 resulted in a decrease of the ratio between inclusion/exclusion of exon 18 in ATXN2 transcripts to 0.26-fold or 0.33-fold (Fig. 7D). Thus, FOX-2 dependent splicing of ATNX2 transcripts is affected by ATXN1 overexpression.

## Discussion

In this study, we functionally analyzed the interaction between ATXN1 and FOX-2 splice variants. The *FOX* genes are highly diverse due to the use of alternative promoters and alternative splicing, and the respective splice variants exhibit nuclear as well as cytoplasmic localization [8], [37]. As mentioned, FOX-2 is highly expressed in the brain and in the cerebellar cortex suggesting high splicing activity in this location [7], [47]. Therefore, it is interesting that FOX-2 localization and splicing activity was affected in the presence of nuclear ATXN1 inclusions, which are detected in post-mortem brain tissue of SCA1 patients and transgenic SCA1 mouse models [22], [24]. Given that the formation of nuclear ATXN1 inclusions is independent of the length of the polyglutamine stretch [25], [43], we did not observe a significant difference in the co-localization of FOX-2 and normal or mutant ATXN1 in the cell lines tested. Moreover, overexpression of normal ATXN1 leads to neurodegeneration in animal models as well, although to a much lesser extent than mutant ATXN1 [34]. Finally, one should bear in mind that quantitative distinctions are not feasible in our cell line models.

Interestingly, defects in the cellular RNA metabolism have been linked to SCA1 pathogenesis. On one hand, ATXN1 itself is a RNA binding protein whose binding to RNA is affected by the length of the polyglutamine stretch [48], and gene products implicated in binding and processing of RNA have been shown to modify neurodegeneration in transgenic SCA1 fly models [33], [34]. On the other hand, complex formation between mutant ATXN1 and the RNA binding motif protein 17 (RBM17) is increased, thereby lowering the complex between normal ATXN1 and capicua [27]. Co-expression of human RBM17 and mutant ATXN1 worsened the ATXN1-induced eye phenotypes in a transgenic SCA1 fly model [27]. Initially, RBM17 was identified by mass spectrometry to be part of the human spliceosome [49], but has been shown to regulate apoptosis through alternative splicing of FAS, too [50]. Interestingly, an interaction between ATXN1 and another splicing factor, U2AF65, has been reported as well [26]. Here, overexpression of normal ATXN1 has an enhancing effect on U2AF65-mediated splicing, whereas mutant ATXN1 has not; potentially due to interference of the expanded polyglutamine stretch with molecular recognition or due to recruitment or trapping of this factor in nuclear inclusion formed by mutant ATXN1 [26]. Moreover, the mRNA export factor TAP/NXF1 has been shown to be recruited into nuclear ATXN1 inclusions [25].

**Figure 8:**
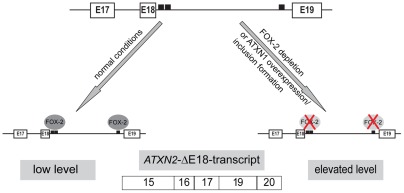
Schematic model of FOX-2 and ATXN1 effects on ATXN2 transcript. The *SCA2* gene bears two putative FOX-binding sites downstream of exon 18 in the ATXN2 transcript as illustrated. Under normal conditions, FOX-2 binding resulted in inclusion of exon 18, whereas depletion of FOX-2 or overexpression of ATXN1 resulted in increased levels of ATXN2 transcripts lacking exon 18.

Another intriguing aspect is that ATXN2, a protein itself involved in the cellular RNA metabolism [44], [51], [52], has been shown to modulate SCA1 pathogenesis, since ATXN2 overexpression enhances, whereas ATXN2 depletion reduces ATXN1-induced toxicity in a fly model [28]. Moreover, nuclear accumulation of ATXN2 was observed in post-mortem brain of a SCA1 patient and correlated to SCA1 pathogenesis [28]. In light of this, the finding that splicing of ATXN2 transcripts is affected by FOX-2 depletion as well as by overexpression of normal and mutant ATXN1 is interesting and it is quite tempting to speculate that alterations in ATXN2 transcripts and their cellular consequences affect SCA1 pathogenesis (Fig. 8). Although we used non-neuronal cell lines such as HEK293T and HeLa cells in our study, the results obtained could be quite relevant for SCA1, since both reproduce molecular mechanisms contributing to SCA1 pathogenesis in the cerebellum [25], [27], [41], [42]. So far, some ATXN2 splice variants have been described that might fulfill different functions [52], [53], [54], e.g. in the central nervous system the ATXN2 full-length transcript is predominantly present in the brain and spinal cord, while expression of a ATXN2 splice variant lacking exon 10 is more prominent in the cerebellum [54]. Therefore, further insight into the cellular function of different ATXN2 splice variants and their regulation and whether and how this relates to mechanisms underlying SCA1 will be an interesting aspect in the future.

On the other hand, these insights might also be valuable with regards to the polyglutamine disorder SCA2, in which ATXN2 represents the causative protein. In this study, we have provided first evidence that both FOX-2 variants directly interact with ATXN2 as well. Thus, it will be interesting to investigate the biological consequence of the ATXN2/FOX-2 interaction and whether mutant ATXN2 has an impact on FOX-2 splicing activity in general and particularly on ATXN2 transcripts *per se*. Notably, we observed an interaction between the predominantly nuclear FOX-2 variant and the ATXN2 region comprising the LSm domain that forms the structural core of spliceosomal small nuclear ribonucleoproteins and is speculated to bind to splicing complexes, implicating ATXN2 *per se* in splicing events [55]. Accordingly, first evidence for a nuclear localization of ATXN2 has been provided [28], [56]. On the other hand, the predominantly cytoplasmic FOX-2 variant was found to interact with the C-terminal region of ATXN2 containing the PAM motif. Several splicing factors were also found to function in the cytoplasm or were detected in stress granules, cytoplasmic sites existing in mammalian cells under various stress conditions that are involved in translational control [57], [58], [59]. In line with these observations, we discovered that FOX-2, like ATXN2, is a component of stress granules. Interestingly, the *Xenopus* homolog of FOX-2, XRbm9, is part of a cytoplasmic polyadenylation complex [60]. Thus, it will be interesting in the future to dissect the cellular functionality of the interactions between the different FOX-2 and ATXN2 variants likely occurring in different compartments.

Splice variants of other polyglutamine proteins have been described at the transcript as well as protein level as pointed out recently by Harris and colleagues [61]. For SCA3, a number of ataxin-3 splice variants have been identified [62], [63], and interestingly, for two variants, differences in their aggregation properties have been demonstrated [61]. Moreover, cell-type-specific alternative splicing of ataxin-6 is likely to contribute to SCA6 pathogenesis [64]. For SCA7, a different cellular localization of a novel ataxin-7 variant in patient tissue has been reported [65]. These findings strongly indicate that alternative splicing of disease-causing genes *per se* is implicated in the pathogenesis of polyglutamine disorders. Since several polyglutamine or ataxia-causing proteins are also connected in a protein-protein interaction network, regulatory effects of splice variants at the protein level are likely to take place as well [16]. Since there is evidence that splicing differs in health and disease, isoform-specific targeting could be a promising therapeutic avenue to approach.

## Materials and Methods

### Plasmids

Plasmid pJET1.2-FOX-2_cyt_ was generated by subcloning a DNA fragment generated via PCR using the oligonucleotide pair FOX-2-s-SalI and FOX-2-as-NotI and human fetal brain library (Clontech; Mountain View, USA) as template. This FOX-2 variant is identical to the FOX-2 transcript variant 1 (ENST00000449924), but contains an additional 32 bp insertion between exon 12 (ENSE00001578447) and exon 13 (ENSE00001611944), which corresponds to an annotated exon (ENSE00001553845) (Fig. S1A; Accession number AB649123, DDBJ). This insertion results in a frame-shift that generates an alternative C-terminal region lacking the hydrophobic PY nuclear localization signal (RF(A/T)PY) that is conserved within the FOX protein family and required for nuclear localization [6], [7]. Confocal microscopy demonstrated that HA-FOX-2_V1_ containing the conserved nuclear localization signal RF(A/T)PY was predominantly located in the nucleus, whereas MYC-FOX-2_cyt_ mainly displayed a cytoplasmic localization (Fig. S1B). For generating the Y2H plasmids pACT-FOX-2_cyt_ and pBTM-FOX-2_cyt_, plasmid DNA pJET1.2-FOX-2_cyt_ was treated with the restriction endonucleases *Sal*I and *Not*I. Afterwards, the respective DNA fragment was isolated and subcloned into the *Sal*I/*Not*I sites of pBTM117c or pACT4-1b, respectively.

Y2H plasmids pBTM-ATXN1-NTQ30, pBTM-ATXN1-NTQ82, pBTM-ATXN1-AXH, and pBTM-ATXN1-CT were created by PCR using primer pairs ATXN1-NT-s-SalI and ATXN1-NT-as-NotI, ATXN1-AXH-s-SalI and ATXN1-AXH-as-NotI, or ATXN1-CT-s-SalI and ATXN1-CT-as-NotI, respectively, and pcDNA1-FLAG-SCA1-Q30 or pcDNA1-FLAG-SCA1-Q82 as template DNA (kind gift of Flaviano Giorgini, University of Leicester). Afterwards, resulting DNA fragments were treated with *Sal*I and *Not*I and subcloned into the *Sal*I/*Not*I sites of pBTM117c. For generating Y2H plasmids pACT-ATXN1-NTQ30, pACT-ATXN1-NTQ82, pACT-ATXN1-AXH or pACT-ATXN1-CT, respective pBTM plasmids were treated with *Sal*I and *Not*I. Resulting DNA fragments were subcloned into the *Sal*I/*Not*I sites of pACT4-1b, respectively.

The mammalian expression construct pCMV-MYC-FOX-2_cyt_ was created by treating plasmid pBTM-FOX-2_cyt_ with *Sal*I and *Not*I. Subsequently, the relevant DNA fragment was purified and subcloned into the *Sal*I/*Not*I sites of the mammalian expression vector pCMV-MYC (Clontech; Mountain View, USA). The mammalian expression plasmid pCMV-HA-FOX-2_V1_ encoding variant 1 of FOX-2 (ENST00000449924) was generated by a multi-step PCR using pCMV-MYC-FOX-2_cyt_ as template. First PCR was performed using primer pair FOX-2-s-SalI and FOX-2-nuc-as1-E12-E13-blunt, which has been designed to contain the last 20 nucleotides of exon 12 in combination with the first 11 nucleotides of exon 13 of the *FOX-2* sequence. In a second PCR step primer pair FOX-2-nuc-s1-E12-E13-blunt, which has been designed to contain the last 10 nucleotides of exon 12 and the first 21 nucleotides of exon 13 of the *FOX-2* sequence, and FOX-2-nuc-as1-NotI was used. Afterwards, both PCR amplicons were purified and mixed to equal concentrations and used as template in a final PCR step with primer pair FOX-2-s-SalI and FOX-2-nuc-as1-NotI resulting in a DNA fragment lacking the 32 nucleotide sequence insertion (ENSE00001553845) present in pCMV-MYC-FOX-2_cyt_. After purification, this DNA fragment was subcloned into vector pJET1.2 to generate plasmid pJET1.2-FOX-2_V1_. Next, plasmid pJET1.2-FOX-2_V1_ was treated with the restriction endonucleases *Sal*I and *Not*I and the corresponding DNA fragment was isolated and subcloned into the *Sal*I/*Not*I sites of the mammalian expression vector pCMV-HA or the Y2H vectors pBTM117c and pACT4-1b, to generate plasmids pCMV-HA-FOX-2_V1_, pBTM-FOX-2_V1_ and pACT-FOX-2_V1,_ respectively.

The mammalian expression construct pCMV-MYC-TIAR (variant 2) was generated by PCR using the oligonucleotide pair TIAR-s-SalI and TIAR-as-NotI and human fetal brain library (Clontech; Mountain View, USA) as template. The amplified DNA fragment was subcloned into vector pCMV-MYC.

The Y2H plasmids encoding different LexA-ATXN2 fusion proteins and the mammalian expression plasmid pCMV-ATXN2-Q22 were described earlier [44], [51], [66]. Mammalian expression plasmids encoding CFP-ATXN1-Q0, CFP-ATXN1-Q30 and CFP-ATXN1-Q82 were a kind gift of C.C. Tsai (Robert Wood Johnson Medical School, New Jersey, USA).

Primers used in this study are listed in Table 1 and constructs were validated by sequencing.

**Table 1:** : Oligonucleotides used in this study.

Oligonucleotides for PCR	sequence (5′-3′) (underlined regions represent introduced restriction sites)
FOX-2-s-SalI	GCGTCGACG ATGGAGAAAAAGAAAATGGTA
FOX-2-as-NotI	ATTTGCGGCCGCTTTA TCAGTAGGGGGCAAATCGGC
FOX-2-nuc-as1-E12-E13-blunt	CATATCCACCATAGAGGTCAGCACCGTAAA
FOX-2-nuc-s1-E12-E13-blunt	TGACCTCTATGGTGGATATGCAGCCTACAG
FOX-2-nuc-as1-NotI	ATTTGCGGCCGCTT TCAGTAGGGGGCAAATCGGC
ATXN1-NT-s-SalI	ATGTCGACA AAATCCAACCAAGAG
ATXN1-NT-as-NotI	TTAGCGGCCGCATCA TTTCATGAAGTAGGG
ATXN1-AXH-s-SalI	ATGTCGACG TCCGTGGCCTCCCCG
ATXN1-AXH-as-NotI	TTAGCGGCCGCATCA CACGGGCTGGCCCTTTTT
ATXN1-CT-s-SalI	ATGTCGACG AGCGAGAACTTCAAC
ATXN1-CT-as-NotI	TTAGCGGCCGCA CTACTTGCCTACATT
TIAR-s-SalI	GCGTCGACG ATGATGGAAGACGACG
TIAR-as-NotI	ATTTGCGGCCGC TCACTGTGTTTGGTAACT

Oligonucleotides for qPCR	sequence (5′-3′)
ATXN1-E8-fw	AGCCATAGCCCGGGCGTGGCCGTGA
ATXN1-E9-rev	CTGGCTGGTTCTCTCCGGAC
ATXN2-E17-18-fw	AGGCGTGCAACCTTTATACC
ATXN2-E18-rev	CTGCTCTATATGTCTTGGCT
ATXN2-E17-19-fw	AGGCGTGCAATACCAAATAT
ATXN2-E19-rev	CTGAGACTGATAATGTGGCA
beta-actin_fw	CGGATGTCCACGTCACACTT
beta-actin_rev	GTTGCTATCCAGGCTGTGCT
B2M_fw	ACTGAATTCACCCCCACTGA
B2M_rev	CCTCCATGATGCTGCTTACA
HPRT_fw	AGGAAAGCAAAGTCTGCATTGTT
HPRT_rev	GGTGGAGATGATCTCTCAACTTTAA
MAP3K7-fw	GGAGCAGTGTGGAGAGCTTG
MAP3K7-E12-rev	CTGATATGACGATCTCAGGG
MAP3K7-E11-13-rev	TGTCCGTTGCCTGTGGTTGC
FOX-2-fw	CCTGGCTTCCCTTACCCTAC
FOX-2-rev	AGCAGGCTGTGCATATCTGT

### Yeast Two Hybrid analysis

Yeast strain L40ccua was co-transformed with the bait constructs pBTM-FOX-2_V1_ or pBTM-FOX-2_cyt_ and the series of pACT-ATXN1 prey constructs, or with the different pBTM-ATXN2 constructs and pACT-FOX-2_V1_ or pACT-FOX-2_cyt_, respectively. Afterwards, transformants grown on SD media lacking tryptophan and leucine were isolated and analyzed for the activity of the reporter genes. For this, yeast cells grown in the respective liquid media were spotted onto solid medium lacking leucine and tryptophan and onto solid medium lacking tryptophan, leucine, uracil and histidine. For analyzing the activity of the *LacZ* reporter gene, cells were spotted onto a nylon membrane (Micron Separations Inc.; Westboro, USA). Growth on plates was analyzed after 3–5 days. For analysis of *LacZ* reporter gene activity, the membrane was incubated in liquid nitrogen and subsequently placed on Whatman paper saturated with X-Gal buffer (phosphate buffer pH 7.0, 0.15% X-Gal, 10 mM DTT) for four to six hours at 37°C.

### Cell cultivation and transfection

HeLa and HEK293T cells (ATCC, Manassas, USA) were cultivated in Dulbecco's modified Eagle medium (Gibco-Invitrogen; Paisly, UK) supplemented with 100 U/ml Penicillin, 100 µg/ml G-Streptomycin (Biochrom; Berlin, Germany) and 10% fetal bovine serum (Biochrom; Berlin, Germany) at 37°C and 5% CO_2_. For transient transfections, cells were grown in 12- or 24-well plates to a confluence of 50–70%. Then, cells were transfected with 1–2 µg of the corresponding mammalian expression plasmids using 3 or 6 µl PEI (Polyethylenimine, Polysciences, Inc.; Warrington, USA) or 2.5 or 5 µl PolyFect (Qiagen; Hilden, Germany), respectively. To allow transient expression of proteins, cells were incubated for 24–48 hours at 37°C and 5% CO_2_.

### Protein lysates and Co-Immunoprecipitation

Co-IP experiments were carried out as described earlier [51], [66]. Briefly, for the association studies between ATXN1 and FOX-2, HEK293T cells overexpressing FLAG-ATXN1-Q30 or non-transfected HEK293T cells were washed once with PBS, treated for 10 minutes with lysis buffer A [10 mM HEPES pH 7.4 (USB corporation; Cleveland, USA), 10 mM NaCl, 3 mM MgCl_2_ (Merck; Darmstadt, Germany), 1 mM DTT (Sigma; St. Louis, USA), 1/7 complete Mini EDTA-free Protease Inhibitor Cocktail (Roche; Mannheim, Germany)], and lysates were passed ten times through a needle. Afterwards, 500 mM NaCl was added, samples were incubated for 20 minutes at 4°C on a rotation wheel and cleared by centrifugation (5 minutes, 5000 rpm, 4°C; rotor: eppendorf FA-45-24-11). Then, the supernatant fractions were transferred to a new tube. The pellet fractions were treated with lysis buffer B [10 mM HEPES pH 7.4, 300 mM NaCl, 20 mM MgCl_2_, 1 mM DTT, 1/7 complete Mini EDTA-free Protease Inhibitor Cocktail, 0.2 U DNase (Sigma; St. Louis, USA)] for 30 minutes at 37°C, cleared again by centrifugation and these fractions were mixed with the initial supernatant fractions. For the association studies between ATXN2 and FOX-2, HeLa cells were washed with PBS, treated with lysis buffer [20 mM Tris-HCl pH 7.4 (Merck; Darmstadt, Germany), 150 mM NaCl (Merck; Darmstadt, Germany), 1 mM EDTA (Sigma; St. Louis, USA), 1% Triton X-100 (Merck; Darmstadt, Germany), 1 µl/10 ml Benzonase (Merck; Darmstadt, Germany), 1/25 complete Protease Inhibitor Cocktail (Roche; Mannheim, Germany)], incubated for 30 minutes on ice, and lysates were cleared by centrifugation (1 minute, 14000 rpm, RT).

For Co-IP experiments 1 mg of each cell lysate was incubated with 4 µl antibody against FOX-2 [mouse anti-RBM9 M01 (Abnova; Taipei City, Taiwan)] or 2 µl rabbit anti-RBM9 (Bethyl Laboratories; Montgomery, USA) and incubated overnight at 4°C. For ATXN1 overexpression experiments, 200 µg cell lysate and 2 µl rabbit anti-RBM9 was incubated overnight at 4°C as well. Then, 30 µl Protein G-conjugated Dynabeads (Dynal-Invitrogen; Paisly, UK) or 15–20 μl IgG-conjugated M-280 Dynabeads (Dynal-Invitrogen; Paisly, UK) were added to lysates and further incubated for three hours. Dynabeads were pulled down magnetically and washed twice in 3% BSA/PBS and twice in PBS. Then, SDS-sample buffer was added to elute bound proteins, and samples were incubated at 95°C for 10 minutes. After separation of proteins by 10% SDS-PAGE and transfer to a PVDF-membrane (Millipore; Billerica, USA) or nitrocellulose membrane (Whatman; Springfield Mill, UK) using a PerfectBlue semidry electroblotter (PeqLAB Biotechnologie; Erlangen, Germany), membranes were incubated overnight with anti-ATXN1 [1∶500, rabbit (Sigma; St. Louis, USA)], anti-ATXN2 [1∶1000, mouse (BD-Biosciences; Franklin Lakes, USA)] or anti-FLAGM2 [(1∶1000, mouse (Sigma; St. Louis, USA)] as primary antibodies. Following incubation with secondary antibodies [1∶10000 POD-conjugated anti-rabbit (Sigma; St. Louis, USA), 1∶10000 POD-conjugated anti-mouse (Sigma; St. Louis, USA)], proteins were visualized using Western Lightning ECL (Perkin Elmer; Massachusetts, USA).

### RNAi experiments

HeLa and HEK293T cells were seeded in 12-well plates in Dulbecco's modified Eagle medium (Gibco-Invitrogen; Paisly, UK) supplemented with 10% fetal bovine serum and incubated for twenty-four hours. Then, 2.4 µl of the respective 20 µM siRNAs [ON-TARGETplus SMARTpool, human RBM9 (FOX-2), ON-TARGETplus Non Targeting Pool; (Dharmacon; Lafayette, USA)] were mixed with 100 µl Dulbecco's modified Eagle medium and 3 µl Lipofectamine RNAiMAX Transfection Reagent (Invitrogen; Paisly, UK) for 20 minutes and subsequently added to the cells. Total RNA was isolated from cells seventy-two hours post transfection using the RNeasy Mini Kit (Qiagen; Hilden, Germany) as recommended by the manufacturer. Concentration of RNA isolates was determined with a Nano Drop ND-1000 Spectrophotometer (NanoDrop Technologies; Wilmington, USA).

### Quantitative real-time PCR

The quantitative real-time PCR (RT-qPCR) was performed as described [56]. Briefly, for reverse transcription, 2 µg of total RNA was mixed with 0.5 µl Oligo-dT_15_-Primer (Promega; Madison, USA), brought to a final volume of 9.5 µl, and kept for 5 minutes at 70°C with a subsequent incubation for 5 minutes at 4°C. Then, 9.4 µl DEPC (Diethylpyrocarbonate)-treated water (Ambion; Austin, USA), 5 µl 5x M-MLV-reverse transcriptase buffer (Promega; Madison, USA), 0.5 µl dNTPs and 0.1 µl M-MLV reverse transcriptase (Promega; Madison, USA) were added, samples were incubated for 1 hour at 42°C, and the reaction was stopped by incubating samples at 65°C for 10 minutes. Then, RT-qPCRs were performed in triplicates in a 10 μl volume and processed with the ABI Prism 7900HT sequence detection system (Applied Biosystems; Foster City, USA). Each reaction contained 50 ng cDNA, 0.25 µmol of the respective primers and 2.5 µl SYBR Green PCR Master Mix (Applied Biosystems; Foster City, USA). As reference genes *ACTB* (β-actin), *HPRT* (hypoxanthine phosporibosyltransferase) and *B2M* (β-2-microglobulin) were used. Fold change was calculated with the ΔΔCt method (User Bulletin #2, Applied Biosystems), and used for calculating the inclusion/exclusion ratio of the analyzed mRNA transcripts. Standard error of the mean was calculated for four independent experiments each with three replicates. For detection of ATXN2 transcripts lacking exon 18 conditions as described above were used, and samples were loaded onto a 3% agarose gel. Primers used are listed in Table 1.

### Confocal microscopy

For localization studies of endogenous FOX-2 levels, HeLa and HEK293T cells were cultivated and fixed as described above. For overexpression experiments, cells were transfected with corresponding constructs, incubated for forty-eight hours, treated with cold methanol (Merck; Darmstadt, Germany) for 1 hour at –20°C, washed with PBS and treated for 30 minutes with 3% BSA/PBS. Then, cells were incubated with primary antibodies [1∶200 mouse anti-ATXN2 (BD-Biosciences; Franklin Lakes, USA); 1∶200 rabbit anti-ATXN2 (Sigma-Aldrich; St. Louis, USA), 1∶200 rabbit anti-RBM9 (Bethyl Laboratories; Montgomery, USA); 1∶200 mouse anti-RBM9 (Abnova; Taipei City, Taiwan); 1∶500 mouse anti-FLAGM2 (Sigma; St. Louis, USA); 1∶500 mouse anti-HA (Roche; Roche; Mannheim, Germany); 1∶500 rabbit anti-MYC (Sigma; St. Louis, USA); 1∶500 mouse anti-MYC (Millipore; Billerica, USA)] for one hour at RT or overnight at 4°C. Afterwards, cells were washed three times with PBS and the respective dye-conjugated secondary antibodies [1∶500 anti-mouse Alexa Fluor488 (Molecular Probes-Invitrogen; Paisly, UK); 1∶500 anti-rabbit Alexa Fluor568 (Molecular Probes-Invitrogen; Paisly, UK); 1∶500 anti-mouse-Cy3 (Jackson Immuno Research; West Grove, USA); 1∶10 HA-Fluorescine (Roche; Mannheim, Germany)] were added for one hour. Nuclei were stained with bisBenzimide [Hoechst (Sigma; St. Louis, USA)] and cells were subsequently washed with PBS and mounted with Fluoromount-G (Southern Biotech; Birmingham, USA). Cells were analyzed using a confocal laser-scanning microscope system LSM700 Imager M2 (Carl Zeiss; Jena, Germany) with oil objectives (Zeiss Plan-NEOLUAR 40x/ 1.3 or 63x/ 1.4 DIC). 8-bit images were taken and processed using ZEN 2009 V5.5 software (Carl Zeiss; Jena, Germany).

## Supporting Information

Figure S1 **FOX-2 splice variants.** (**A**) (Upper panel) Schematic view of the FOX-2 splice variants FOX-2_V1_ and FOX-2_cyt_. Sequence of the additional exon within FOX-2_cyt_ is indicated. (Lower panel) Alignment of the C-terminal region of FOX-2_V1_ and FOX-2_cyt_. Insertion of an additional exon (ENSE00001553845) causes a frame-shift resulting in a different C-terminal ending. Yellow highlights identical amino acids. (**B**) Localization of FOX-2_V1_ and FOX-2_cyt_. HeLa cells were transiently transfected with expression plasmids pCMV-HA-FOX-2_V1_ or pCMV-MYC-FOX-2_cyt_ and incubated for twenty-four hours. Afterwards, cells were fixed and proteins were stained with HA-Fluorescine (upper panel) or anti-MYC antibody (lower panel). Bars represent 20 µm.
(TIF)

Figure S2 **FOX-2 accumulates in nuclear ATXN1 inclusions in HEK293T cells.** HEK293T cells expressing normal and mutant ATXN1 were fixed forty-eight hours post transfection. Endogenous level of FOX-2 was visualized using a specific antibody (Abnova). Nuclei were stained using Hoechst. Bars represent 20 µm.
(TIF)

Figure S3 **FOX-2 localizes to stress granules under stress conditions.** (**A**) HeLa cells were transiently transfected with plasmids pCMV-HA-FOX-2_V1_ or pCMV-MYC-FOX-2_cyt_. Twenty-four hours post transfection, cells were left untreated (upper panel) or exposed to 0.5 mM arsenite for one hour (lower panel). Then, proteins FOX-2_V1_ and FOX-2_cyt_ were visualized with HA-Fluorescine or with an anti-MYC antibody as indicated in Material and Methods. ATXN2 as stress granule marker protein was co-stained using an anti-ATXN2 antibody (Sigma). (**B**) For studying endogenous FOX-2 localization, HeLa cells left untreated (upper panel) or exposed to 0.5 mM arsenite for one hour (middle and lower panel) were fixed and stained with antibodies against FOX-2 (Bethyl), ATXN2 (BD-Biosciences) or TIAR. Nuclei were stained using Hoechst. Bars represent 20 µm.
(TIF)
