# Supplementary figures and images for: FOX-2 Dependent Splicing of Ataxin-2 Transcript Is Affected by Ataxin-1 Overexpression
Source: PLoS One. 2012 May 30;7(5):e37985. doi: 10.1371/journal.pone.0037985 (PMC3364202; doi:10.1371/journal.pone.0037985)

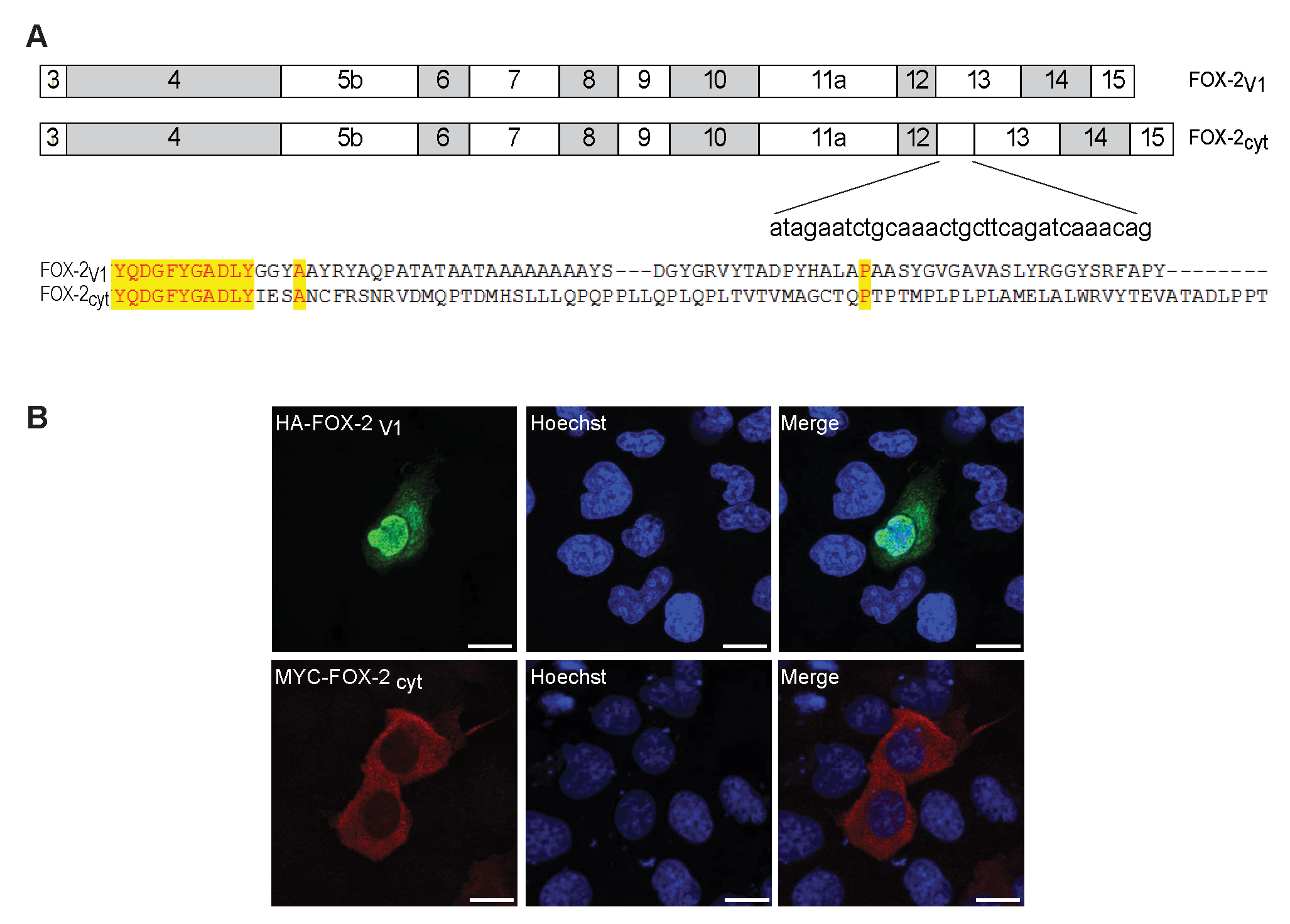

Supplement: Figure S1 — FOX-2 splice variants. (A) (Upper panel) Schematic view of the FOX-2 splice variants FOX-2V1 and FOX-2cyt. Sequence of the additional exon within FOX-2cyt is indicated. (Lower panel) Alignment of the C-terminal region of FOX-2V1 and FOX-2cyt. Insertion of an additional exon (ENSE00001553845) causes a frame-shift resulting in a different C-terminal ending. Yellow highlights identical amino acids. (B) Localization of FOX-2V1 and FOX-2cyt. HeLa cells were transiently transfected with expression plasmids pCMV-HA-FOX-2V1 or pCMV-MYC-FOX-2cyt and incubated for twenty-four hours. Afterwards, cells were fixed and proteins were stained with HA-Fluorescine (upper panel) or anti-MYC antibody (lower panel). Bars represent 20 µm. (TIF) [file pone.0037985.s001.tif]

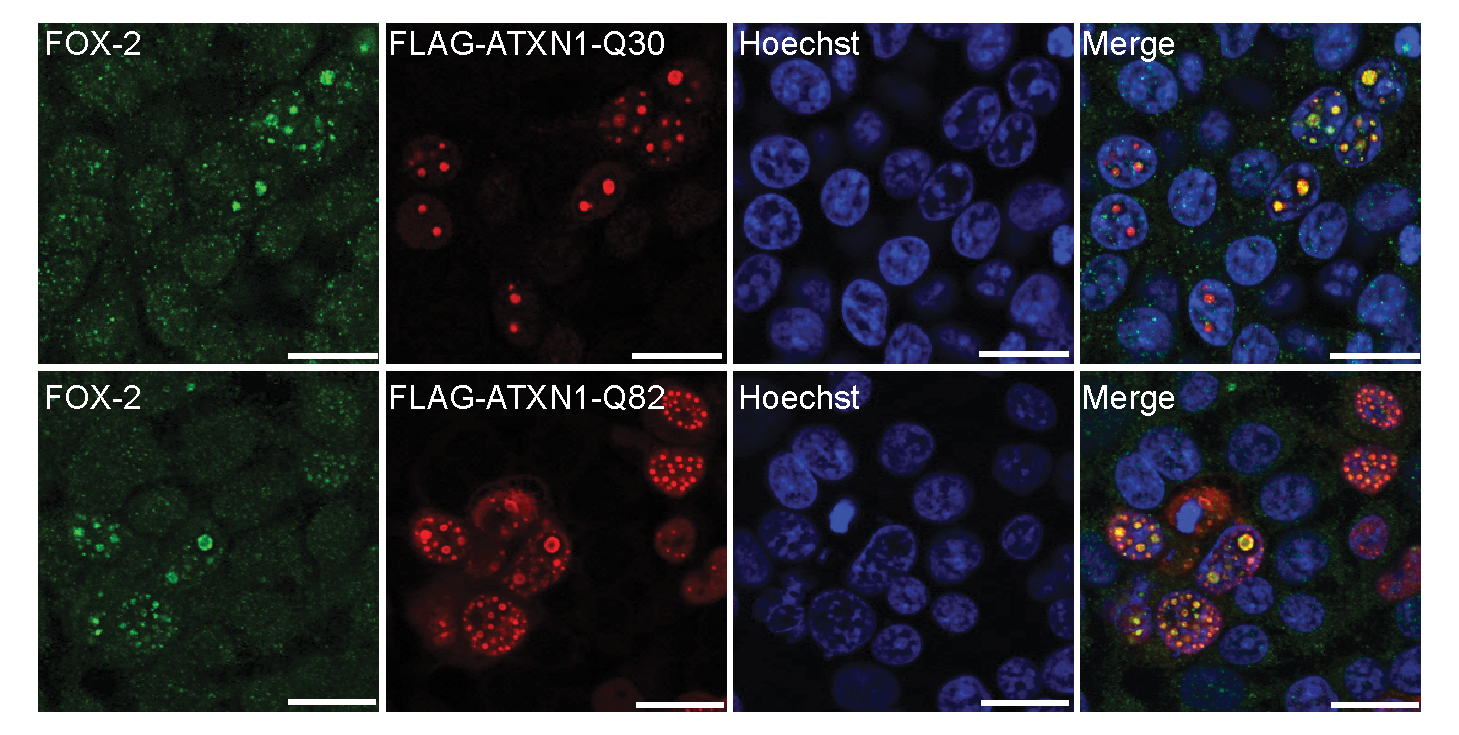

Supplement: Figure S2 — FOX-2 accumulates in nuclear ATXN1 inclusions in HEK293T cells. HEK293T cells expressing normal and mutant ATXN1 were fixed forty-eight hours post transfection. Endogenous level of FOX-2 was visualized using a specific antibody (Abnova). Nuclei were stained using Hoechst. Bars represent 20 µm. (TIF) [file pone.0037985.s002.tif]

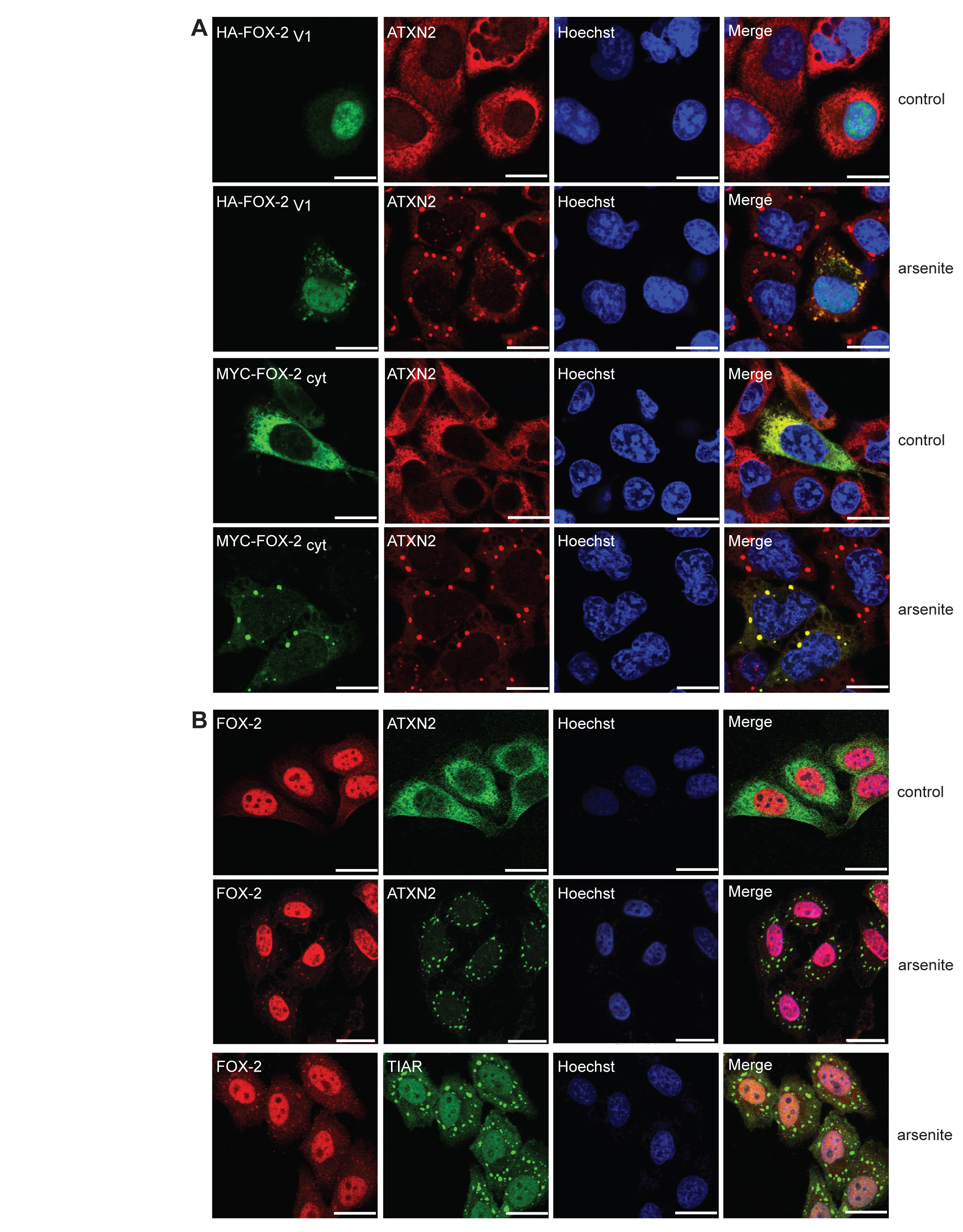

Supplement: Figure S3 — FOX-2 localizes to stress granules under stress conditions. (A) HeLa cells were transiently transfected with plasmids pCMV-HA-FOX-2V1 or pCMV-MYC-FOX-2cyt. Twenty-four hours post transfection, cells were left untreated (upper panel) or exposed to 0.5 mM arsenite for one hour (lower panel). Then, proteins FOX-2V1 and FOX-2cyt were visualized with HA-Fluorescine or with an anti-MYC antibody as indicated in Material and Methods. ATXN2 as stress granule marker protein was co-stained using an anti-ATXN2 antibody (Sigma). (B) For studying endogenous FOX-2 localization, HeLa cells left untreated (upper panel) or exposed to 0.5 mM arsenite for one hour (middle and lower panel) were fixed and stained with antibodies against FOX-2 (Bethyl), ATXN2 (BD-Biosciences) or TIAR. Nuclei were stained using Hoechst. Bars represent 20 µm. (TIF) [file pone.0037985.s003.tif]
